# Evidence for gill slits and a pharynx in Cambrian vetulicolians: implications for the early evolution of deuterostomes

**DOI:** 10.1186/1741-7007-10-81

**Published:** 2012-10-02

**Authors:** Qiang Ou, Simon Conway Morris, Jian Han, Zhifei Zhang, Jianni Liu, Ailin Chen, Xingliang Zhang, Degan Shu

**Affiliations:** 1 Early Life Evolution Laboratory, School of Earth Sciences and Resources, China University of Geosciences, Beijing 100083, China; 2 Department of Earth Sciences, University of Cambridge, Downing Street, Cambridge CB2 3EQ, UK; 3 Early Life Institute and Key Laboratory of Continental Dynamics, Northwest University, Xi'an 710069, China; 4 Chengjiang Fauna National Geopark of China, Chengjiang 652500, China

## Abstract

**Background:**

Vetulicolians are a group of Cambrian metazoans whose distinctive bodyplan continues to present a major phylogenetic challenge. Thus, we see vetulicolians assigned to groups as disparate as deuterostomes and ecdysozoans. This divergence of opinions revolves around a strikingly arthropod-like body, but one that also bears complex lateral structures on its anterior section interpreted as pharyngeal openings. Establishing the homology of these structures is central to resolving where vetulicolians sit in metazoan phylogeny.

**Results:**

New material from the Chengjiang Lagerstätte helps to resolve this issue. Here, we demonstrate that these controversial structures comprise grooves with a series of openings. The latter are oval in shape and associated with a complex anatomy consistent with control of their opening and closure. Remains of what we interpret to be a musculature, combined with the capacity for the grooves to contract, indicate vetulicolians possessed a pumping mechanism that could process considerable volumes of seawater. Our observations suggest that food captured in the anterior cavity was transported to dorsal and ventral gutters, which then channeled material to the intestine. This arrangement appears to find no counterpart in any known fossil or extant arthropod (or any other ecdysozoan). Anterior lateral perforations, however, are diagnostic of deuterostomes.

**Conclusions:**

If the evidence is against vetulicolians belonging to one or other group of ecdysozoan, then two phylogenetic options seem to remain. The first is that such features as vetulicolians possess are indicative of either a position among the bilaterians or deuterostomes but apart from the observation that they themselves form a distinctive and recognizable clade current evidence can permit no greater precision as to their phylogenetic placement. We argue that this is too pessimistic a view, and conclude that evidence points towards vetulicolians being members of the stem-group deuterostomes; a group best known as the chordates (amphioxus, tunicates, vertebrates), but also including the ambulacrarians (echinoderms, hemichordates), and xenoturbellids. If the latter, first they demonstrate that these members of the stem group show few similarities to the descendant crown group representatives. Second, of the key innovations that underpinned deuterostome success, the earliest and arguably most seminal was the evolution of openings that define the pharyngeal gill slits of hemichordates (and some extinct echinoderms) and chordates.

## Background

Molecular data [1,2] have radically reconfigured many aspects of metazoan phylogeny, and in doing so have effected a renaissance in this area of evolutionary biology that has uncovered many hitherto unsuspected relationships. An unavoidable drawback, however, is that typically the extant forms are highly derived and disparate with the result that the natures of common ancestors are often matters for unconstrained speculation. In principle, however, by providing direct access to members of stem groups of particular phyla (or other major clades), the fossil record can provide insights into the early divergence of major groups and hence the construction of their respective bodyplans [3]. In reality the available fossil record remains problematic, because although in many cases stem-group representatives have proved of great value in documenting the nature of transitional forms, in the Cambrian there is a plethora of seemingly enigmatic taxa. Interpretations of such taxa can be so disparate that it is sometimes questioned whether what is known of these fossils makes them capable of serving any useful phylogenetic role [4,5]. A key area of dissent is the recognition of homologous structures. This, combined with often apparently bizarre combinations of character states, has generated controversy rather than consensus. Despite being widespread and often showing excellent soft-bodied preservation, the enigmatic vetulicolians [6-13] exemplify this difficulty.

This group is united by a distinctive bodyplan. This consists of a prominent anterior unit, ranging from oval to quadrate in shape, and possessing a wide oral opening. Attached to this unit is a segmented tail with a terminal anus. Despite a strikingly arthropod-like arrangement, none of the known taxa (including *Vetulicola *[6], *Ooedigera *[13], *Pomatrum *[14], *Xidazoon *[15], *Didazoon *[16], *Beidazoon *[17], *Yuyuanozoon *[18], and *Banffia *[10]) has yielded evidence of either molting or convincing remains of cephalic structures and/or jointed limbs. Nevertheless, vetulicolians continue to be compared to ecdysozoans [19] as far flung as the lobopodians [20], arthropods [6,10,21], and kinorhynchs [7,22,23]. A radical alternative has been to assign them to the deuterostomes, as either members of a stem group [13,16,17,24] or some sort of tunicate [18,25,26]. The crux of the problem revolves around the nature of prominent lateral structures, but to date there is neither agreement as to their original configuration nor function. Identifications range from midgut glands [27] to respiratory organs, but in the latter case interpretations are as disparate as a direct comparison to crustacean branchial chambers [12,20,21,28] as against pharyngeal gill slits [13,16,17,24]. Resolution of this divergence of interpretations would be possible if exquisitely preserved material were available that would allow not only a complete anatomical description but one that can be combined with a plausible functional analysis. Here, we demonstrate that the lateral structures of *Vetulicola *form an integrated complex and we present evidence for what we interpret to be a series of five unequivocal openings. We further propose that these served to expel seawater from the interior, which previously had been filtered so as to convey food first to dorsal and ventral gutters and ultimately to the posterior gut.

## Results

### Functional morphology of gill slits in *Vetulicola*

Material of *Vetulicola *is abundant, but relatively few specimens are sufficiently well preserved and/or orientated so as to reveal all the necessary details. Importantly the anterior unit is usually filled with sediment, which appears to have been introduced post mortem. This, combined with the fact that it can be removed by mechanical preparation, means that in addition to the customary external views, details of the inner configurations are also available. Any interpretation, therefore, depends crucially on the relative orientation and taphonomy of individual specimens. Viewed from the exterior the anterior region of *Vetulicola *consists of effectively four plate-like units that are covered by a thin membrane. These plates meet along both the dorsal and ventral midlines, but are laterally separated by prominent grooves that extend the entire length on either side. The margins of both grooves are darker in color. We suggest the darker color represents somewhat thicker material that would have served as reinforcement. This feature is most notable at the five semicircular regions (the lappets). Both margins of the groove form an overhang. Below we review evidence that these grooves could expand and it seems likely that this recessed area was composed of more delicate tissue. At both ends of the anterior unit the groove tapers to a fine termination, but the intervening length shows various complexities on both the outer and inner surfaces. Below, we justify why we believe that this region houses perforations that link the interior of the animal to the exterior, and then go on to argue that these are homologous to the canonical pharyngeal gill slits of deuterostomes. We are mindful that observation and interpretation (functional and phylogenetic) need to be kept separate, and thus at this stage reference to gill slits should be taken as a descriptive shorthand.

**Figure 1:**
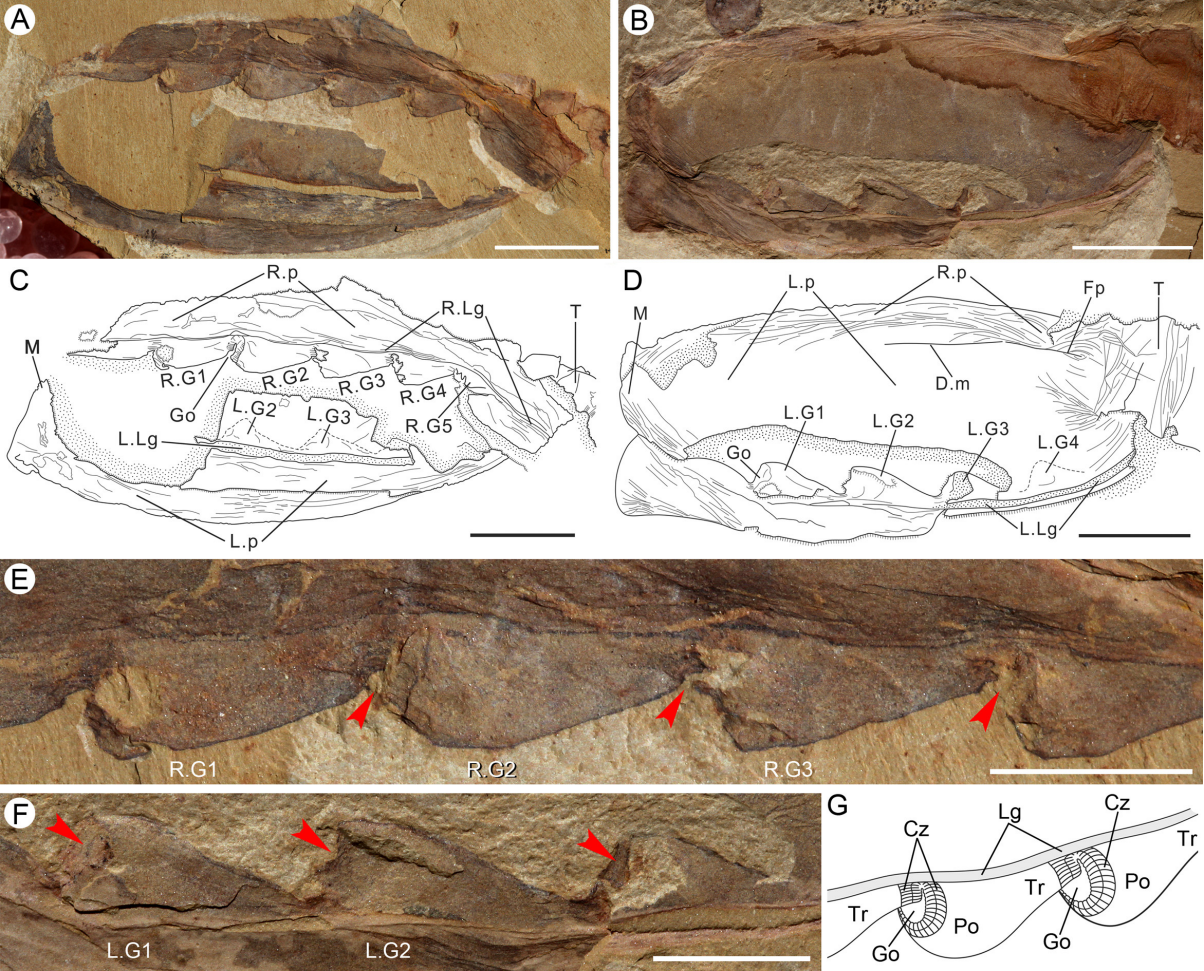
**Gill anatomy of *Vetulicola rectangulata *from Yunnan, China**. **(A,B) **Latero-obliquely compressed specimens ELEL-EJ081561A and ELEL-EJ080255A with internal gill openings. **(C,D) **Interpretative line drawings of (A) and (B), respectively. **(E,F) **Details of right gill slits 1 to 4 and left gill slits 1 to 3 in (A) and (B), respectively; internal openings (arrowheads; notably right gill 2 in (E)) are surrounded by a zone of striations and folds. **(G) **Schematic reconstruction of *Vetulicola *gill system (viewed from the interior) with plates and lappets flanking the lateral groove removed for clarity, showing two adjacent gill slits with anteriorly positioned apertures. Abbreviations: Cz, concentric zone; Dm, dorsal midline; Fp, fin-like process; Go, gill opening; Lp, left plate; LG1-4, left gill slits 1 to 4; LLg, left lateral groove; M, mouth; Po, gill pouch; RLg, right lateral groove; Rp, right plate; RG1-5, right gills 1 to 5; T, tail; Tr, gill trough. Scale bars: 1 cm in (A) to (D); 5 mm in (E) and (F).

Viewed from the exterior, the initial length of the groove shows a uniform expansion before abruptly widening in order to accommodate the first opening. This arrangement is effectively repeated another four times before the groove narrows to its final termination at the posterior margin. When viewed from the interior the groove can be seen to stand proud of the surrounding region and this suggests that it was suspended into the anterior cavity, but with expanded areas forming an imbricated array of five pouch-like structures, each one of which then tapers posteriorly (Figure 1A-G). Along the midline of each pouch there is usually a narrow ridge (Additional file 1). The five openings that define the actual gill slits are defined by oval perforations in the body wall. Their association with the lateral grooves results, however, in a more complex anatomy, which can only be resolved in exceptional material that exposes both the internal and external views. Given that the interior of the animal is usually clogged with sediment, this, combined with the fact that the openings had a finite thickness, means that the openings are visible from the interior. Therefore, if the sediment that accumulated in the groove is removed, the relationship between the opening and the surrounding areas becomes more obvious.

**Figure 2:**
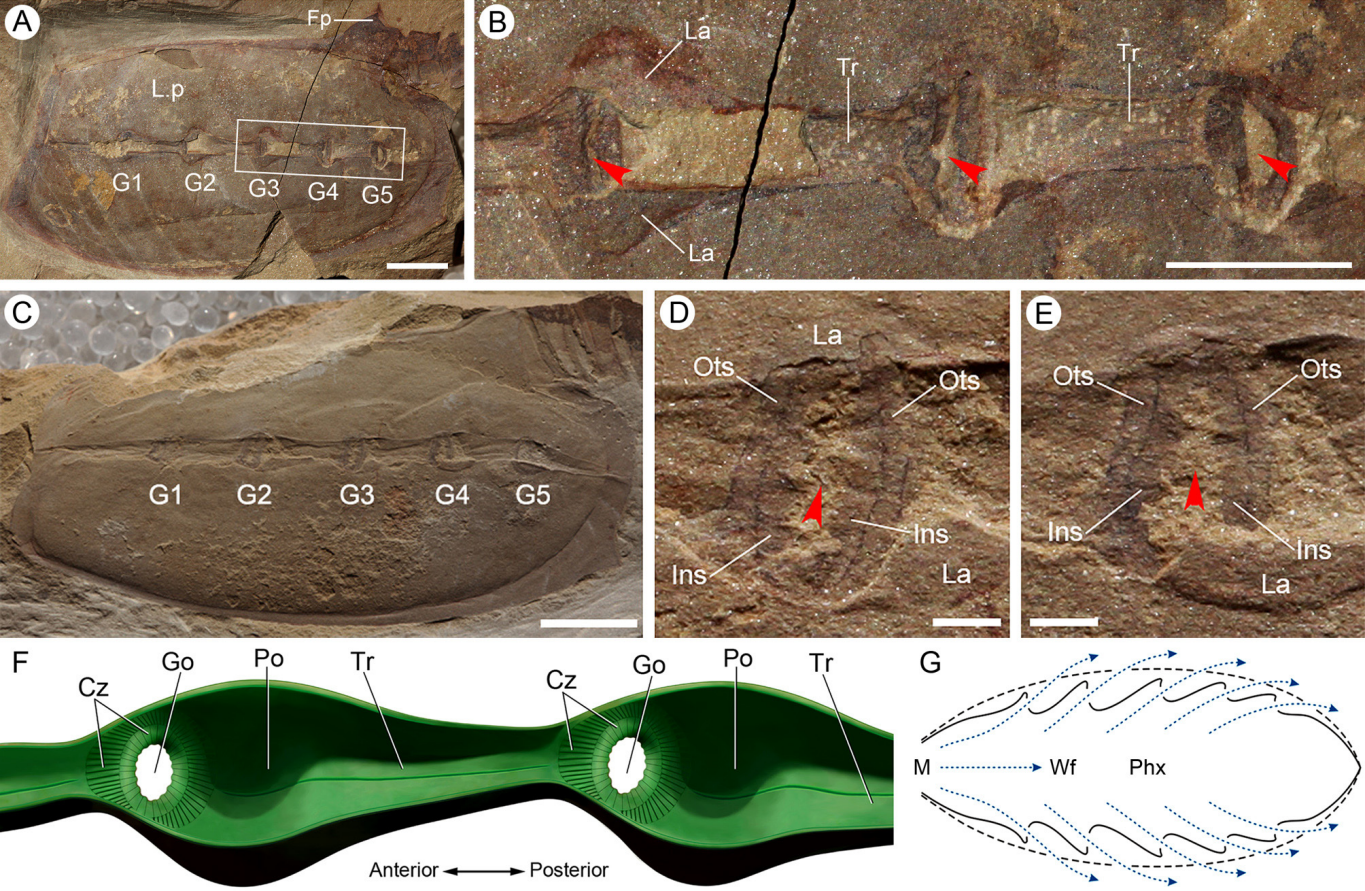
**Gill system and reconstruction in *Vetulicola rectangulata***. **(A) **Laterally compressed specimen ELI-1037A, viewed from the exterior. **(B) **Enlargement of left gill slits 3 to 5 in (A) (boxed area), showing gill openings (arrowheads; notably gill 3) surrounded by a folded and striated zone. **(C) **Laterally compacted specimen ELI-1121A. **(D,E) **Close-up images of left gill slits 3 and 4, respectively, both showing a concentric zone that surrounds the gill pore; a narrow dark line separates the concentric zone into an inner and an outer section; both units possess radial striations. **(F) **Three-dimensional reconstruction of two adjacent gill slits (external view) with plates, lappets, and overhanging margins of the groove removed for clarity; note the gill pores are surrounded by a concentric zone and located anterior of each pouch, which posteriorly constricts into a trough. **(G) **Schematic horizontal section through the lateral groove of *Vetulicola*, with inferred ventilation of feeding-respiratory currents (dotted blue lines); lateral grooves (dashed black lines) can be either open or closed. Abbreviations: Cz, concentric zone; Fp, fin-like process; G, gill slit; Go, gill opening; Ins, inner section; La, lappet; Lp, left plate; M, mouth; Ots, outer section; Po, gill pouch; Tr, trough. Scale bars: 1 cm in (A), (C), (h); 5 mm in (e), (f), (i); 1 mm in (j), (k).

Viewed from the interior, each opening is directed anteriorly and so orientated subparallel to the lateral wall (Figures 1A-G and 2G). What is interpreted as the inhalant aperture is surrounded by striated units (Figure 1E), which we propose served as flexible membranes. Viewed from the exterior the shape of the opening is more or less slit-like and typically is filled with sediment (Figure 2A-E; Additional file 1). From this perspective the margins of the aperture form an integral part of the groove, but in contrast to its general smoothness are defined by zones of either folds or dark striations. On the posterior margin of each aperture a transverse ridge with relatively prominent striations occurs (Additional file 1). To the immediate anterior of the aperture an expanded region typically consists of low folds, which impart a characteristic scalloped appearance (Figure 2B,D-F). The closeness of the folds and variation in the width of this region suggest it could contract and so they may have helped to control the size of the opening. Such functioning as a sort of valve is also supported by measurements of the diameter of the opening versus the width of the pouch. Whereas the overall correlation coefficient (r^2 ^= 0.6086 to approximately 0.7532; see Additional files 2 and 3) is consistent with isometric growth, the residuals may represent degrees of apertural dilation.

In specimens where the grooves remain filled with sediment the openings are apparently isolated, but in this aspect they provide details of the region from which the water exited. Typically the lappets obscure the upper and lower margins, but when removed by mechanical preparation the actual apertures (typically these are filled with sediment) have an oval outline. The margin that defines the aperture is irregular and bluntly dentate, while the surrounding area consists of two distinct concentric units that are separated by a narrow dark partition (Figure 2B,D-F; Additional file 1). Both units possess radial fibers, but those of the outer zone seem to be finer and more numerous. In some specimens the inner unit appears to be crumpled and this, together with the dentate margin (Figure 2D-F), suggests that at least partial closure was possible. The common feature of radial fibers surrounding the aperture, whether viewed from the interior or exterior, suggests that these structures defined the apertural walls and most likely served a structural or possibly muscular role. Their dark color may also indicate vascularization.

### Pumping dynamics

**Figure 3:**
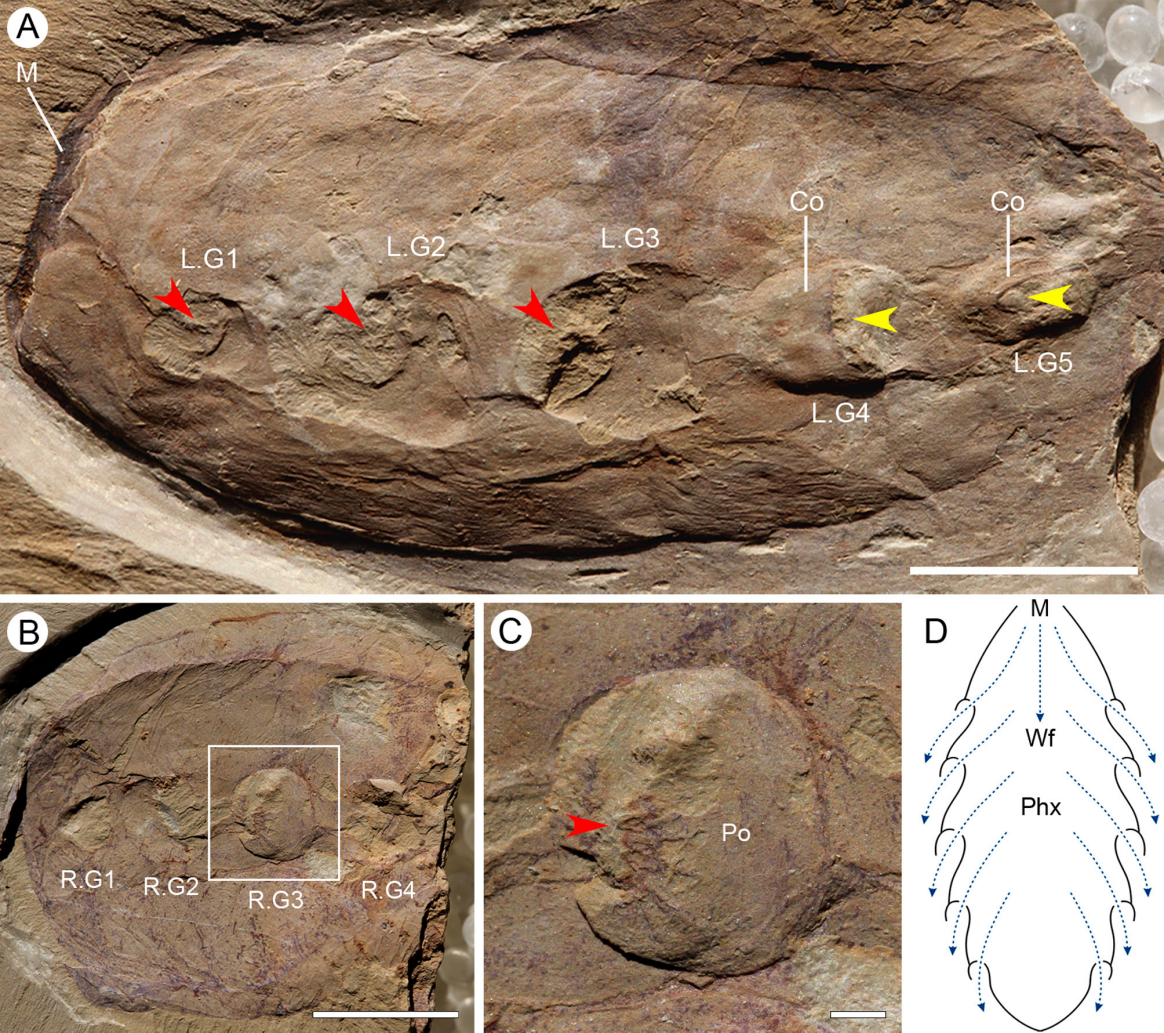
**Gill structures of *Didazoon haoae***. **(A) **Laterally compressed specimen ELI-2010A (external view); internal openings (inhalant apertures) are revealed (red arrowheads) in left gills 1 to 3 by removal of gill cowls; intact left gill slits 4 and 5 show cowls and exhalant apertures (yellow arrowheads). **(B) **Laterally compressed specimen ELI-JS1001A, viewed from the interior. **(C) **Close-up image of right gill slit 3 in (B) (boxed area); note the oval inhalant aperture (arrowhead) surrounded by plate-like structures and radiating strands. **(D) **Schematic horizontal section through the lateral midline of *Didazoon*, with inferred ventilation of feeding-respiratory currents (dotted blue lines). Abbreviations: Co, cowl-like structure; LG1-5, left gills 1 to 5; M, mouth; Phx, pharynx; Po, gill pouch; RG1-4, right gills 1 to 4; Wf, water flow. Scale bars: 1 cm in (A), (B); 1 mm in (C).

**Figure 4:**
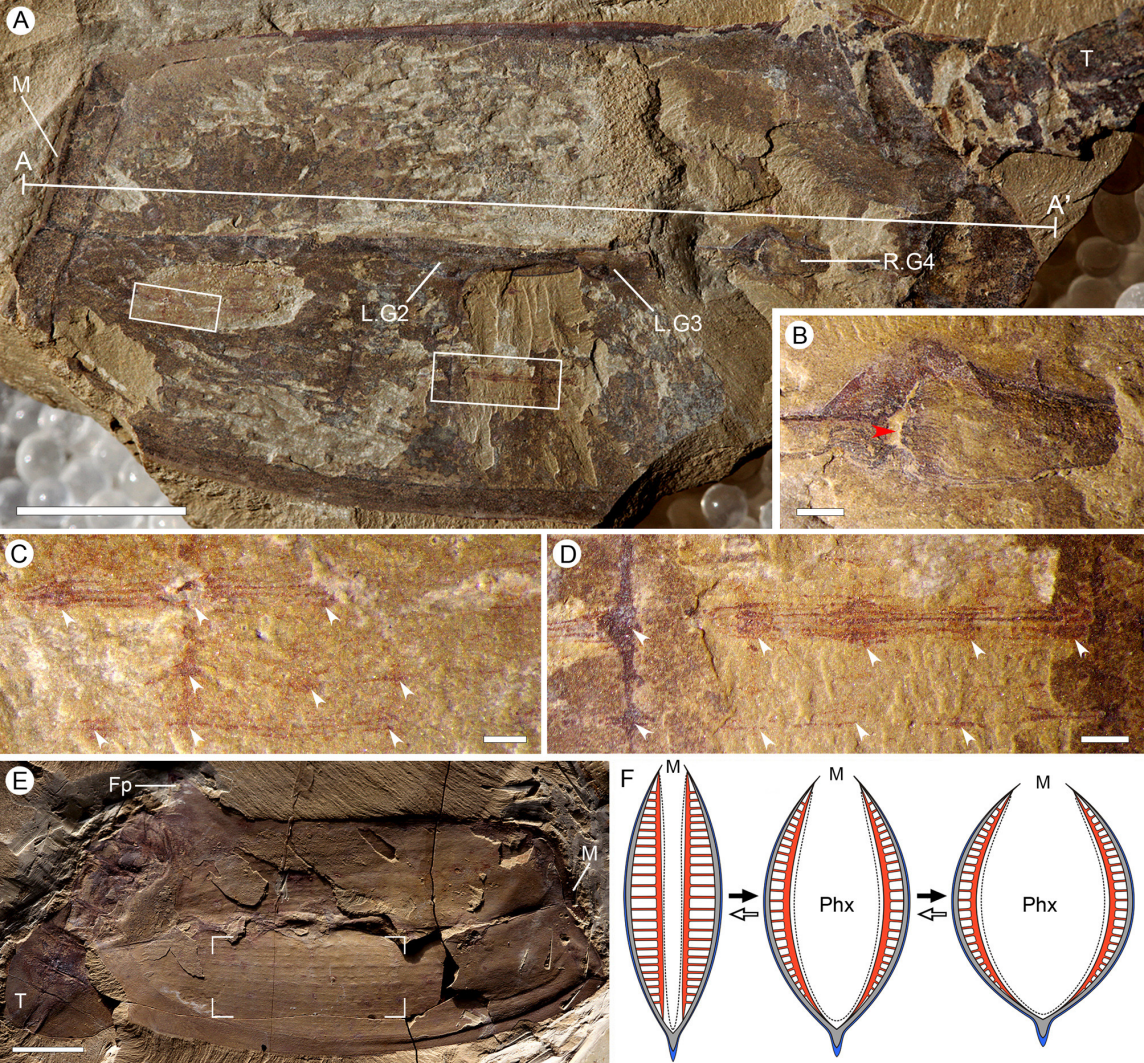
**Gill anatomy and body-wall musculature of *Vetulicola rectangulata***. **(A) **Laterally compressed specimen ELI-SJ0605A, with preserved muscular fibers beneath the plate-like unit; note also the tightness of the lateral groove. **(B) **Internal view of right gill slit 4 in (A); note the internal opening (red arrowhead) surrounded by striations. **(C,D) **Enlargement of boxed areas in **(A) **at left and right side, respectively, showing fibrous structures interpreted as longitudinal muscles, defined by diagenetic iron minerals; note their location beneath the plates and arrangement as continuous bundles (approximately six fibers per bundle) extending almost the entire length of the anterior section; note also the equidistant expansions (arrowheads), interpreted here as corresponding to a column of horizontal muscles. **(E) **Specimen ELI-0000306, showing inferred muscular imprints represented by longitudinal structures in the anterior section (notably the focused area). **(F) **Reconstructed horizontal section (membrane in blue, plates in grey, musculature in red), equivalent to section A-A' in (A), demonstrating inferred dynamics of pharyngeal dilation, governed by the collective action of the longitudinal muscle fibers and their horizontal derivatives. Abbreviations: Co, cowl-like structure; Fp, fin-like process; LG, left gill slit; M, mouth; Phx, pharynx; RG, right gill slit; T, tail; Wf, water flow. Scale bars: 1 cm in (A), (E); 1 mm in (B) to (D).

The demonstration of unequivocal lateral perforations (Figures 1, 2, 3) is consistent with water entering the animal via its oral opening and subsequently being expelled along either side of the animal (Figures 2G and 3D). Irrespective of whether, as we argue below, the apertures are homologous with the gill slits of deuterostomes, the inferred water flow invites a consideration as to whether the mechanism was effectively passive, relying on currents induced by ciliary action, or forced by active pumping. It is the case that a wide range of suspension-feeding deuterostomes, notably enteropneusts, most urochordates (apart from salps; see below), amphioxus and by implication extinct gill-bearing echinoderms, employed ciliary mechanisms, and this would accord with a consensus that among the deuterostomes the primitive mode of feeding was passive. While this cannot be ruled out for *Vetulicola*, we suggest that several features of the body of this animal are consistent with a more active mode of water flow. In our view, the most compelling observation revolves around the arrangement of the anterior unit and especially the longitudinal grooves, which we propose played a key role. While evidently having the same composition as the rest of the anterior body wall, the grooves appear to have been more flexible [28]. This is particularly evident from the range of configurations that vary from specimens where the groove is effectively closed (Figure 4A) to others where either margin is widely separated (Additional file 1). In the latter the floor of the groove displays a narrow median depression that anteriorly diverges into a Y shape (Additional file 1); this we presume would have been used to accommodate any extension. Construction of the anterior part of the vetulicolian body as four semi-independent plates with junctions along the dorsal and ventral midlines, combined with dilation of the grooves, would permit expansion of the internal cavity and so allow ingress of seawater. Correspondingly, contraction of the anterior unit would serve to expel the water through the gill slits. Dorsal and ventral sutures [16], that defined the meeting points of the plates, may also have been zones of relatively weakness and by rotating would have contributed to the expansion and contraction of the anterior unit. We have considered whether any other function for these grooves is more plausible. If, for example, vetulicolians ingested large prey then such dilation might assist capture, but overall the evidence for suspension feeding seems stronger.

A second reason to support the idea of a more dynamic mode of suspension feeding in *Vetulicola *concerns its overall morphology, which is indicative of relatively active swimming. In principle this too would have assisted with intake of water. The final point, which is less secure, concerns the relatively small size of the apertures as against the inferred capaciousness of the interior cavity (as judged by the sediment infill). While this involves a number of imponderables, the ratio of aperture diameter to interior volume suggests that ciliary flow alone may not have been sufficient to remove the water efficiently.

We suggest that swallowing and the subsequent expulsion of water most likely would have been coordinated, with control of both the size of the oral opening and lateral apertures. If it is accepted that pumping of water was indeed an active process, then the alternating dilation and contraction most likely would have been mediated by a musculature. Exceptionally preserved material reveals a series of longitudinal structures connected by vertical units (Figure 4A,C-E; see also [22]). Similar, albeit less organized, soft tissues have been tentatively compared to either internal appendages or muscles [22]. The structures described here are provisionally identified as body-wall musculature. We make here the assumption that originally they consisted of horizontal and longitudinal components so that their contractions would determine the volume of the anterior cavity (Figure 4F). Alternative possibilities, not least these structures might represent some part of the filtration unit, certainly merit attention. Nevertheless, the evidence already mentioned in support of a dilatory capacity by the grooves would imply the existence of some sort of antagonistic system. Our reconstruction of this muscular activity, which was possibly assisted by an inherent elasticity of the body wall, provides a working model.

### Gill slits in other vetulicolian taxa

**Figure 5:**
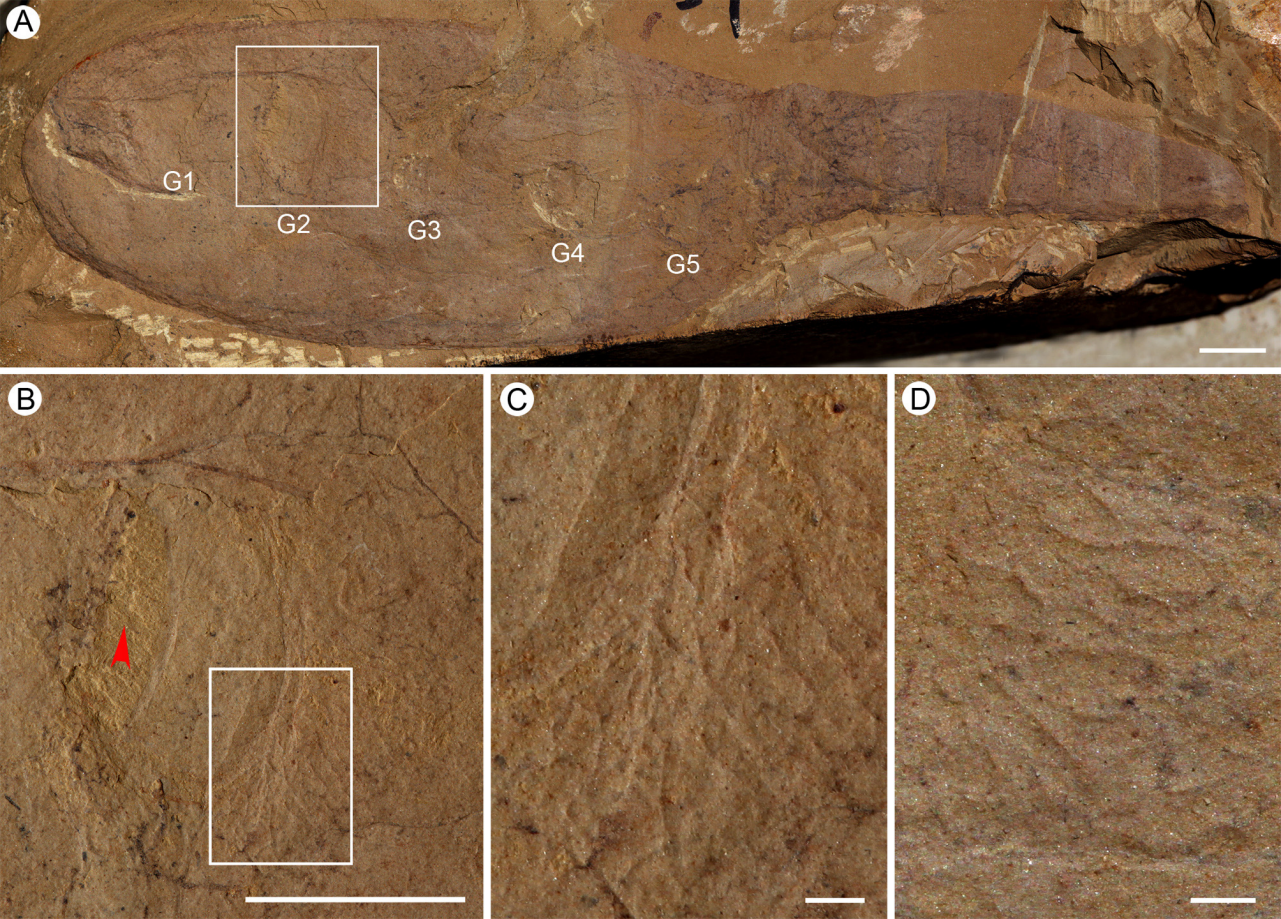
**Gill structures of *Yuyuanozoon magnificissimi *from Yunnan, China**. **(A) **Holotype, complete specimen CFM00059. **(B) **Enlargement of left gill slit 2 in (A) (box), showing the actual slit (arrowhead) and posterior radiating structures. **(C,D) **Close-up images of areas indicated in (B) (box) and that posterior of gill slit 3 in (A), respectively, both showing probable interconnection of radiating structures; these structures appear to represent underlying narrow grooves which may have functioned as part of the vascular system (less likely external gill filaments, as interpreted in [18]). G1-5, gills 1 to 5. Scale bars: 1 cm in (A), (B); 1 mm in (C), (D).

Taxa such as *Didazoon, Pomatrum, Ooedigera, Xidazoon *and *Yuyuanozoon *are all characterized by five sets of paired openings located on the anterior section of the body (Additional file 3). New material of *Didazoon *confirms the earlier supposition [16] that the five external openings, which evidently have a less complex anatomy than that of *Vetulicola*, are connected to the interior (Figure 3). They are, however, directly comparable since in interior aspect the gill slits formed expanded pouch-like structures and as in *Vetulicola *they also have anteriorly directed inhalant apertures. Plate-like structures associated with these apertures are consistent with controlled closure, while radiating strands adjacent to the aperture suggest the chamber had some contractibility (Figure 3C). In addition, while *Didazoon *lacks the prominent grooves of *Vetulicola*, each pouch projects posteriorly as a shallow tube-like structure that imbricates with the succeeding pouch. We suggest that this arrangement represents the precursor of the grooves. Assuming an evolutionary trend among the vetulicolians towards greater motility, then a comparison between *Didazoon *(and morphologically similar taxa) and *Vetulicola *suggests that the changes in anatomy, including a well-developed articulatory tail and more complex apertural arrangements, reflect such a transition. Of these various vetulicolians *Yuyuanozoon *is, however, unusual because behind each aperture there are a series of radiating structures (Figure 5). These have been interpreted as external filaments and as such might have served a respiratory role. They are, however, quite distinct from the filamentous structures seen in association with the openings of *Vetulicola *([16] and Additional file 4). Our re-examination suggests that they actually represent narrow grooves located on the interior of the body wall but if they were vascular they could have played an important role in gas exchange.

### Feeding in a capacious pharynx

**Figure 6:**
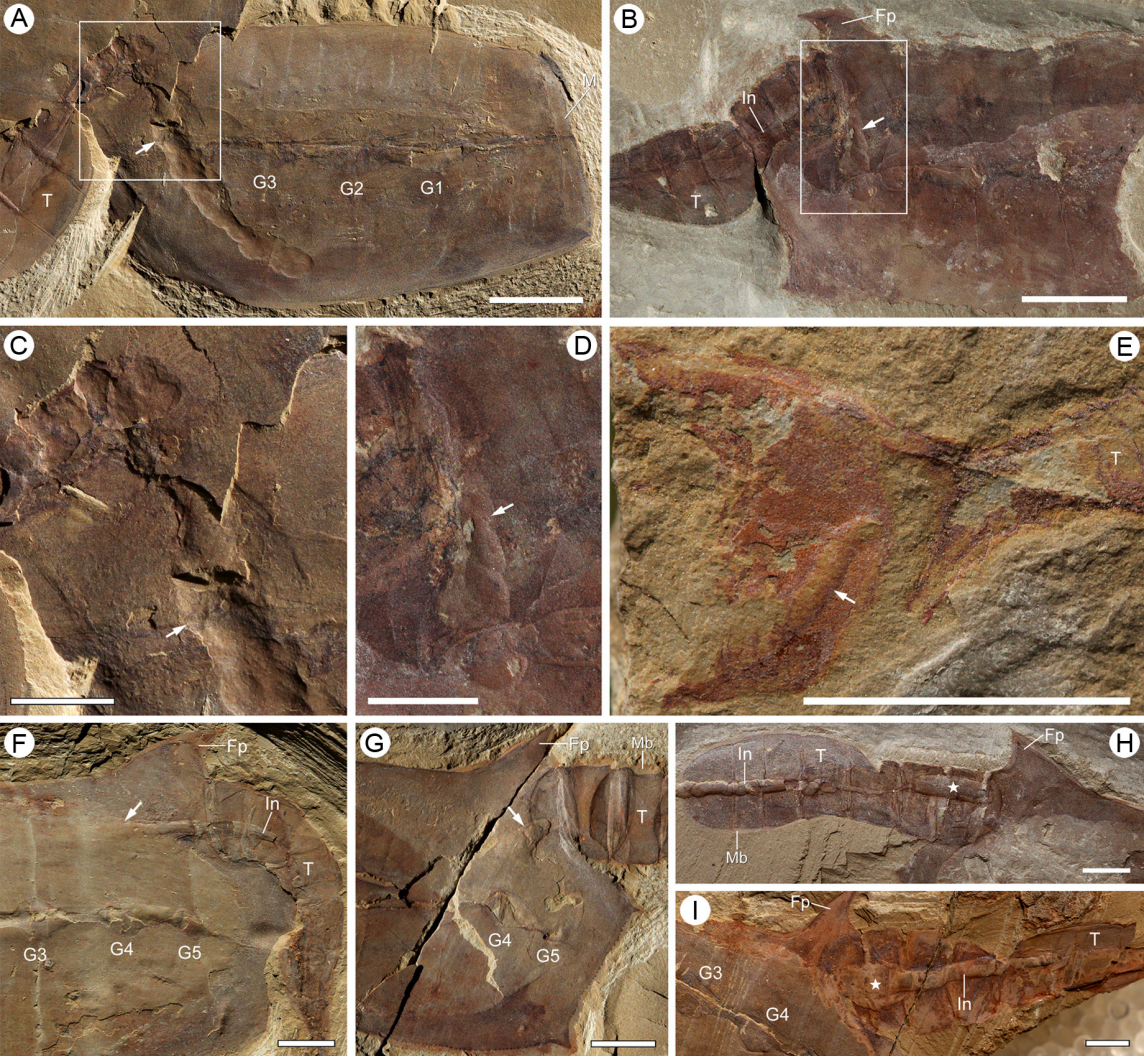
**Evidence for the presence of a pharynx and dorsal/ventral feeding gutters in vetulicolians**. **(A) ***Vetulicola monile *(ELI-SJ1221A) and **(B) ***Vetulicola rectangulata *(ELI-SJ1168A), showing remains of food boluses (arrowed) located along a ventral gutter in posterior region of the pharyngeal cavity. **(C,D) **Close-up images of boxed areas in (A) and (B), respectively; note that posteriorly the food boluses are in continuity with the intestine within the tail. **(E) ***Heteromorphus longicaudatus *(ELI-SJ1247), showing food remains (arrowed) along the ventral side. **(F,G) ***V. rectangulata*, specimens ELI-SS004A and SS002A, both showing food remains (arrowed) and rearward constriction of pharynx, indicating site of a putative esophagus. **(H,I) ***V. rectangulata*, specimens ELI-EJ05832 and ELI-1033A, both showing an expansion (starred) in proximal region of the intestine, possibly equivalent to a stomach. Abbreviations: Fp, fin-like proboscis; G, gill slit; In, intestine; M, mouth; Mb, membrane; T, tail. Scale bars: 1 cm in (A), (B), (E); 5 mm in (C), (D), (F) to (I).

The primary purpose of the vetulicolian openings appears to have been the expulsion of what must have been voluminous quantities of seawater. Given the inferred capaciousness of the anterior cavity it is possible that at least some gas exchange occurred across the internal membranes. Respiratory exchange might also have occurred across the openings themselves and this would be consistent with their inferred vascularization. In this context, therefore, it seems reasonable to refer to these structures as pharyngeal gill slits. In contrast to the other vetulicolians *Vetulicola *is interpreted as an active swimmer, principally because of a propulsive tail [24] and compressed cross-section [16] that would have been relatively streamlined and unstable if positioned on the sea floor. However, as with the related taxa *Vetulicola *we follow previous workers and presume it to have been a suspension feeder. Food interception and collection is thus inferred to be a mucociliary process and is consistent with a spacious pharyngeal cavity that tapered posteriorly towards the opening of the gut (Figure 6F,G). In this scenario once swallowed it seems reasonable to suppose that the seawater was intercepted by a system of internal baffles (most likely ciliated) that served to extract the suspended food particles. This process must have been closely integrated with the expulsion of the seawater through the openings. While the gut is most obvious in the posterior tail (Figure 6H,I), an anterior extension has been identified on the dorsal side (Figure 6F; see also [22]). Its identification as part of the intestine [22] is, however, unlikely because corresponding boluses also occur on the ventral side (Figure 6A-E). The fact that this arrangement is seen in several vetulicolian taxa, including the relatively distantly related *Heteromorphus *(Figure 6E), is consistent with this ventral disposition being original. Although no single specimen shows food material on both dorsal and ventral sides, all such examples are extremely rare and may represent moribund animals whose feeding processes were disrupted. In any event, this suggests that once captured, and depending on whether initial interception was either above or below the gill line, food particles were then transported towards either the dorsal or ventral midline. We suggest that these housed gutters that channeled the food towards the more posterior intestine.

## Discussion and Conclusions

### Vetulicolians as members of the stem-group deuterostomes

**Figure 7:**
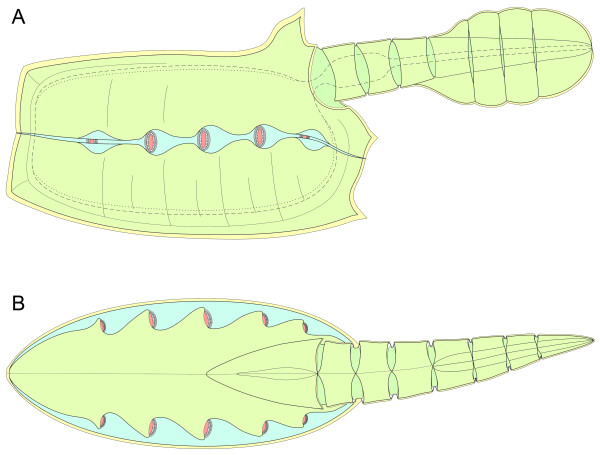
**Schematic reconstruction of *Vetulicola rectangulata***. **(A) **Lateral view. Lappets of left gill 2 to 4 removed to reveal gill openings and surrounding features; pharynx and alimentary canal denoted by dashed line; dorsal and ventral food grooves denoted by dotted lines; gill slits colored in pink. **(B) **Dorsal view. Semidiagrammatical image depicting the internal arrangement of the gills. The diagram is principally to show the overall arrangement of the gills with respect to the rest of the body. Gill morphology is more complex than the diagramatic depiction provided here, and is illustrated in detail in other Figures.

With the information now at hand concerning the complex vetulicolian lateral structures (Figure 7) it appears that their proposed similarities to any known arthropod respiratory structure, and specifically the branchial chambers of a crustacean [12,20,21,28] are not convincing. Even if one was to argue that vetulicolians were still arthropods it is far from clear how any known arthropod gill or respiratory organ could be transformed into the structures observed in the vetulicolians. Despite the strikingly 'phyllocarid-like' appearance of *Vetulicola *[6], the related taxa are much less arthropod like. So too no known bivalved arthropod from the Cambrian provides an obvious link to *Vetulicola*. Finally, our understanding of Cambrian arthropod phylogeny now seems to be reaching a broad consensus [29,30] and in which none of the vetulicolians could be readily accommodated.

A second possibility is that while it is evident that *Vetulicola *and related taxa form a coherent clade, broadly united in possessing a bipartite body with the anterior bearing a series of five perforations on either side, their wider relationships among the Metazoa are simply unresolvable. Given that a number of Cambrian groups such as the nectocariids [31,32] and amiskwiids [33] remain in phylogenetic limbo (but see [34,35]), the only option might be to add the vetulicolians to this roster and continue our efforts to find even better preserved material and/or related taxa that might serve to pinpoint their relationships.

There is, however, a third possibility, that is vetulicolians are some sort of deuterostome. The evidence we present of active pharyngeal pumping might invite comparison to advanced members of this clade, but because of the distinctive bodyplan of the vetulicolians we suggest that this functional feature re-evolved in groups such as the gnathosomes with which, apart from the pharyngeal openings, they share no significant features. We prefer to argue that the vetulicolians are members of the stem-group deuterostomes [13,16,17,24]. Crucial in this regard is the interpretation of the laterally located openings, which as pharyngeal gill slits are taken to be diagnostic of the deuterostomes [36]. Other than what at best seem to be remote analogies in the form of the minute anterior pores in the meiofaunal gastrotrichs [37] no phylum appears to possess comparable structures. On the assumption, therefore, that these openings are not convergent, then the deuterostome status of the vetulicolians means that the spacious anterior would be homologous with the pharynx and the lateral perforations are genuine gill slits.

This conclusion invites brief consideration of three inter-related questions: (a) what implications might the proposed homologies of the gill slits have on other features of the vetulicolians in the context of a deuterostome affinity? (b) What bearing do vetulicolians have on our understanding of deuterostome evolution, not least with respect to the chordates, ambulacrarians and xenoturbellids? And (c) what light might other Cambrian deuterostomes throw on the early evolution of this group?

Our aim here is as much to pose these questions as it is to arrive at unequivocal conclusions. Central to any argument is the recognition of homology. Patterson [38,39] crystallized the discussion by proposing that homology might be recognized in terms of similarity, conjunction and most importantly congruence. Similarity stands on the shakiest ground given that while clearly these are lateral perforations located on the anterior, these proposed gill slits find no exact counterpart in any other deuterostome. However, pharyngeal openings show a remarkable diversity within the deuterostomes, not least among some of the extinct echinoderms [40]. Their possession is, however, universally regarded as a synapomorphy of the deuterostomes [36]. In addition, developmental data are consistent with the earliest deuterostome gill slits being derived from endodermal pockets [36] and in principle originally these might have been little more than pores. In the case of Patterson's [38,39] criterion of what he termed conjunction we can at least argue that (a) vetulicolians do not possess any other structures that are more plausibly identified as gill slits, and (b) that their location on the anterior section is consistent with this identification. By congruence Patterson [38] meant a test for homology based 'on the equivalence of homology and synapomorphy' so that the proposed homology is consistent with the other homologies that are employed to define a monophyletic group. First, we can observe that beyond the deuterostomes no other group of metazoans has an array of lateral openings and as noted despite their undoubted homology [36] they still show an enormous range of anatomical configurations. What then of the relationship of this homology to others that might pin down more precisely where vetulicolians fall in the deuterostomes? The range of phylogenetic conclusions that have already been proposed [22] underlie the difficulty in finding other homologies that one might employ in the use of Patterson's criterion of congruence. In the absence of molecular data and the relative unlikelihood of identifying vetulicolian embryos indicating the fate of the blastopore, very few characters serve to unite the deuterostomes as a whole [41]. Elsewhere we have suggested that vetulicolians had an endostyle and a mesodermal endoskeleton [16], but the evidence is somewhat circumstantial. It is, however, also worth drawing attention to Romer's prescient suggestion [42] that an ancestral form had a bipartite form of a 'somatic' unit with musculature and a 'visceral' component with gill slits. Romer applied this concept to the chordates, but we have argued that his 'dual animal' hypothesis is just as consistent when applied to the vetulicolian bodyplan [16].

If one accepts vetulicolians as deuterostomes then this leads to the question of their possible relationships to the other major groups within this superclade. As already noted we argue that a position within the stem-group deuterostomes is the best phylogenetic solution, but as is often the case in such problematic animals from the Cambrian the correct identification of homologies is crucial. In this context the case of the putative cephalopod *Nectoccaris *[31,32,34,35] provides a useful parallel. Thus any attempt to link the vetulicolians to one or other group within either the ambulcrarians or chordates is largely frustrated not only by the paucity of obvious homologies, but in addition evidence for radical reorganization of some bodyplans (notably echinoderms [43]) as well as both molecular [44] and fossil [45] data that suggest other groups (notably amphioxus) are more or less degenerate. Nowhere is this more evident than in the xenoturbellids (and acoelomorph flatworms) which now appear to be massively simplified deuterostomes (now comprising the Xenocoelomorpha) and the sister group to the ambulacrarians [46]. As such they are uninformative as to either the appearance of the most primitive deuterostomes (let alone bilaterians [47]) or by implication the putative position of the vetulicolians.

Finally, we can ask as to whether, apart from *bona fide *chordates and the like [24,45], are there other Cambrian animals that might be informative with respect to early deuterostome evolution? Foremost in this regard are the coeval yunnanozoans, which elsewhere we have argued [16,24,48] provide a link not only between the vetulicolians but possibly also with representatives of the stem-group chordates [45]. As with the vetulicolians their distinctive bodyplan has led to a diversity of opinions as to their exact placement, but a general consensus places them in the deuterostomes [16,20,24,48]. While a key piece of evidence is the series of external filamentous gills, especially in *Haikouella *[48], unequivocal evidence of pharyngeal openings in any yunnanozoan appears to be wanting.

The results we report here have a bearing on the yunnanozoans in as much as just as the vetulicolians have been compared to arthropods, so too Bergström [19] agrees with earlier observations [24,49] that yunnanozoans have a segmented cuticular surface, at odds with their interpretation as myomeres [48]. This feature, however, need not equate with either an arthropodan or ecdysozoan relationship; nor do we accept Bergström's [19] interpretation of a specimen described by us earlier [16] as evidence for molting. The evidence for a cuticular surface suggests that at least some members of the stem-group deuterostomes possessed a hardened, albeit non-mineralized, exterior. Elsewhere, a remnant of this feature has been tentatively identified in the stem-group chordate *Pikaia *[45].

To conclude, in our view the demonstration that the anterior lateral structures in *Vetulicola *not only have a complex configuration but also unequivocally include perforations is consistent with this animal and related taxa belonging to the deuterostomes. So too the identification of a capacious pharyngeal cavity lined with ventral and dorsal food grooves is consistent with the deuterostome bodyplan and the likely origin of the lateral openings so as to allow exit of seawater.

Deuterostome disparity, both between and within the xenoturbellids, ambulacrarians and chordates, is considerable. Despite now largely secure molecular phylogenies a stumbling block has been how best to envisage the common ancestor. There seems little evidence to suggest that the vetulicolians can be interpreted as the sister group of any of the major groups of deuterostome. There is, for instance, no evidence that the gill slits emptied into a common atrium or otherwise collected the water and expelled it via a single opening. In this and other respects vetulicolians differ from the tunicates [25]. There is, however, one point of comparison that may be relevant. While nearly all tunicates [50] are suspension feeders, among the pelagic representatives the salps are exceptional in employing a muscular pumping [51]. While this is associated with a form of jet propulsion [52], the antagonistic system that involves the tunic and the possibility that this pumping system may have originated in the context of filtration [53] invites analogies with *Vetulicola*. This analogy is, of course, with respect to the dynamics of pumping and given the large and evidently propulsive tail there is no suggestion *Vetulicola *employed any sort of jet propulsion.

We suggest that despite uncertainties the anatomical evidence presented is consistent with *Vetulicola *and its allies as being deuterostomes. While aware that the disparity among the crown-group deuterostomes makes comparisons with the vetulicolians problematic, we are unable to identify any character that would secure the place of this Cambrian group within either the ambulcrarians or chordates. We suggest that the vetulicolians are more likely to be members of the stem-group deuterostomes. If our interpretation of the vetulicolians as being among the earliest deuterostomes is correct then this has a number of important implications. First, it is consistent with the suggestion that such ancestral deuterostomes were not sessile, but free-living animals [13,16,54,55] with an expanded pharyngeal cavity. We take this feature, along with five pairs of gill slits and dorsal and ventral feeding gutters, to represent the primitive condition, and is consistent with the supposition that a filter-feeding pharynx had evolved in the last common ancestor of all deuterostomes [36,56,57]. In addition, we conclude that at least in *Vetulicola *active pharyngeal pumping had evolved, and presumably it arose independently of the system seen in the salps and also more advanced gnathostomes.

Even making allowances for loss and simplification, as well as once popular comparisons (such as the hemichordate stomochord to chordate notochord [58]) very few anatomical homologies serve to unite all the deuterostomes. In this latter respect the pharyngeal openings are a key character. For example, in his proposal that suspension feeding was a feature to emerge very early in the history of deuterostomes, Cameron [57] remarked how 'Clearly the evolution of slits in the pharynx is one of the most important events in the evolution of the deuterostomes'. In our view their identification in the vetulicolians supports the idea that they helped to open the doors to deuterostome diversification, first in terms of effectiveness of feeding and subsequently efficiency of respiratory exchange [17]. Elaborations in the form of the gill slits in both the ambulacrarians and chordates are marked by convergences [41], but in their various configurations they presumably helped to contribute to the success of this phylum. So too we see evolutionary innovation in terms of the disposal of the water, such as the subsequent development of an atrium, and arrangement of food grooves to a single ciliated tract either ventrally (enteropneusts) or dorsally (amphioxus and tunicates). Much of subsequent deuterostome evolution appears to have been driven by ecologies that either favored sessility, with the reduction (as in the pterobranchs) or ultimate loss (as in the echinoderms) of gill slits, or increased motility. The latter may have ultimately led to the transformation of the dorsoposterior segmentation seen in the yunnanozoans [24,48] into a myotomal arrangement with the corresponding evolution of the notochord in the chordates [45]. In conclusion, calls that more data be collected to help secure phylogenetic arguments [4,5] can now be met on the basis of exceptional material. On this basis we argue that the vetulicolians provide an important benchmark in the documentation of the emergence of the deuterostomes.

## Methods

A total of 480 specimens of various vetulicolian taxa (10 species referred to 7 genera), recovered from five localities (Erjie, Sanjie, Jianshan, Tanglipo, and Maotianshan sections) at more or less coeval horizons in the Heilinpu Formation (Lower Cambrian, approximately 520 million years ago), Yunnan, southern China, were prepared and analyzed. About 50 three-dimensionally preserved specimens were 'dissected' to reveal internal anatomy. Details were analyzed using a Zeiss Stemi-2000C microscope (Jena, Germany).

Specimens were reposited in the Early Life Institute (ELI), Northwest University, Xi'an, the Early Life Evolution Laboratory (ELEL), China University of Geosciences, Beijing, China, and the Chengjiang Fauna Museum, Chengjiang Fauna National Geopark, Kunming (CFM), China.

## Competing interests

The authors declare that they have no competing interests.

## Authors' contributions

QO, DS, SCM and JH contributed to this work equally. All authors participated in analysis of vetulicolian specimens and discussion of results. All authors read and approved the final manuscript.

## Supplementary Material

Additional file 1 **Gill structures of *Vetulicola rectangulata *from Yunnan, China**. **(A,B) **Laterally preserved specimens ELEL-SJ101975A and ELEL-EJ080158A, respectively. **(C,D) **Close-up images of the boxed area in (A) and interpretative camera lucida drawing, respectively, showing gill openings, lateral groove, groove floor, Y-shaped medial gutters, transverse ridge posterior of the gill pore, lappets, and their spatial arrangement relative to the adjacent plates. **(E,F) **Close-up images of boxed area in (B) and interpretative camera lucida drawing, respectively, showing dilated lateral groove, groove floor, Y-shaped medial gutters, gill openings, lappets, and their spatial relationship. Abbreviations: Cz, concentric zone that surrounds the gill opening; G1-5, gills 1 to 5; Go, gill opening; La, lappet; Po, gill pouch; Rg, ridge; Se, sediment fill; Tf, trace fossil (unconnected to specimen); Tr, trough; Ys, Y-shaped structure. Scale bars: 1 cm in (A), (B); 5 mm in (C) to (F). Click here for file

Additional file 2 **Linear statistics of gill geometry of *Vetulicola***. **(A-E) **Linear regression of gill pouch width on gill slit diameter (gills 1 to 5, respectively), measured from 130 well-preserved gills in 37 specimens of *Vetulicola rectangulata *(data source: Additional file 3) using PAST software [59], showing the correlation between gill slit diameter and gill pouch width (r: correlation coefficiency). Click here for file

Additional file 3 **Morphometrics of vetulicolian gills**. Morphometrics of vetulicolian gills given in mm. Click here for file

Additional file 4 **Gill filaments in *Vetulicola cuneata *from Yunnan, China**. **(A) **Laterally preserved specimen ELI-0000216. **(B) **Close-up image of the boxed area in (A), displaying tufts of filaments lining the surface of the gill pouch (viewed from the exterior). Transverse, slit-like gill opening denoted by arrowhead. Abbreviations: Fi, filaments; G1-5, gills 1 to 5; La, lappet; M, mouth; T, tail; Tr, trough. Scale bars: 1 cm in (A), (B); 5 mm in (C) to (F). Click here for file
