# Supplementary figures and images for: Evidence for gill slits and a pharynx in Cambrian vetulicolians: implications for the early evolution of deuterostomes
Source: BMC Biol. 2012 Oct 2;10:81. doi: 10.1186/1741-7007-10-81 (PMC3517509; doi:10.1186/1741-7007-10-81)

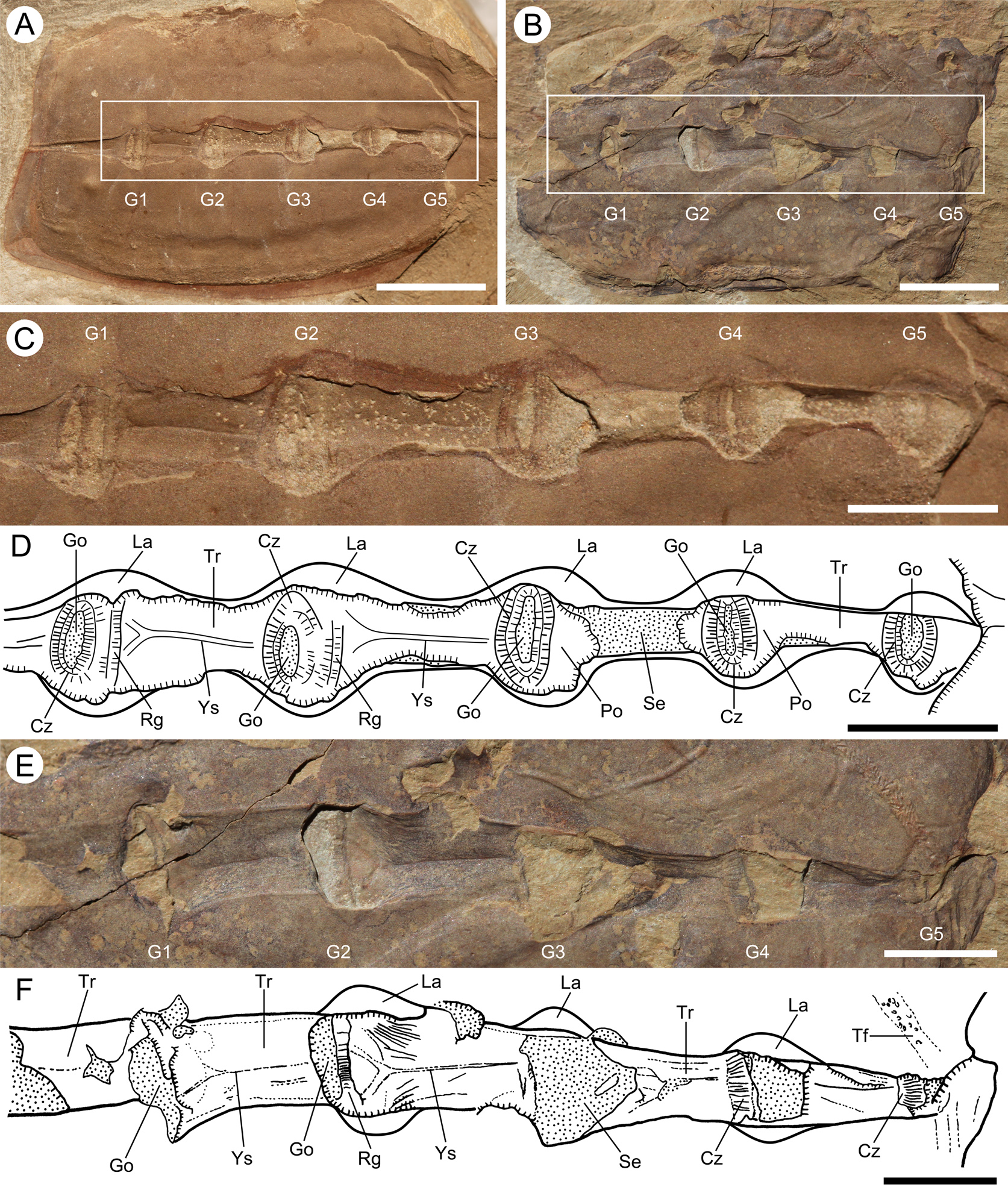

Supplement: Additional file 1 — Gill structures of Vetulicola rectangulata from Yunnan, China. (A,B) Laterally preserved specimens ELEL-SJ101975A and ELEL-EJ080158A, respectively. (C,D) Close-up images of the boxed area in (A) and interpretative camera lucida drawing, respectively, showing gill openings, lateral groove, groove floor, Y-shaped medial gutters, transverse ridge posterior of the gill pore, lappets, and their spatial arrangement relative to the adjacent plates. (E,F) Close-up images of boxed area in (B) and interpretative camera lucida drawing, respectively, showing dilated lateral groove, groove floor, Y-shaped medial gutters, gill openings, lappets, and their spatial relationship. Abbreviations: Cz, concentric zone that surrounds the gill opening; G1-5, gills 1 to 5; Go, gill opening; La, lappet; Po, gill pouch; Rg, ridge; Se, sediment fill; Tf, trace fossil (unconnected to specimen); Tr, trough; Ys, Y-shaped structure. Scale bars: 1 cm in (A), (B); 5 mm in (C) to (F). [file 1741-7007-10-81-S1.JPEG]

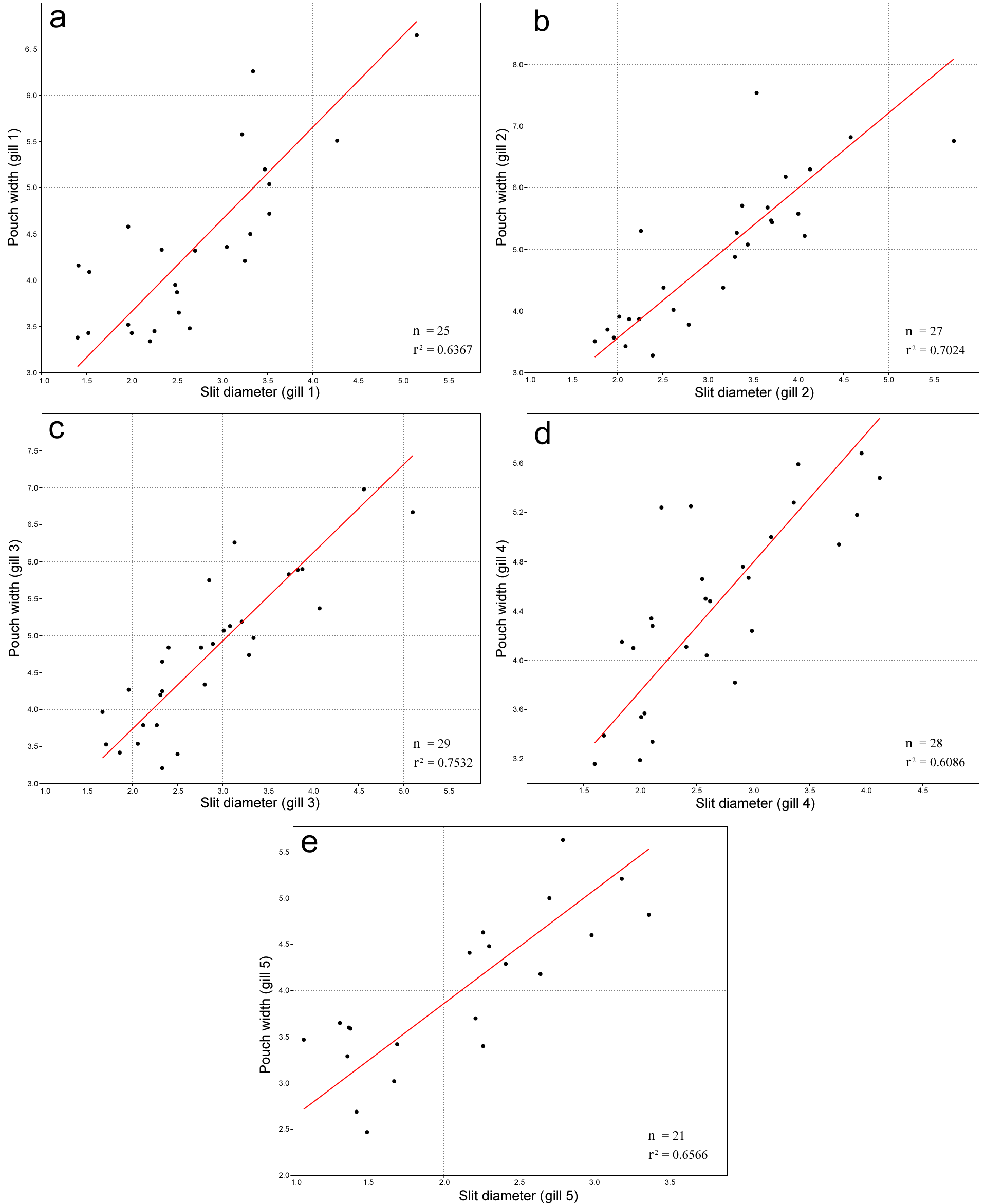

Supplement: Additional file 2 — Linear statistics of gill geometry of Vetulicola. (A-E) Linear regression of gill pouch width on gill slit diameter (gills 1 to 5, respectively), measured from 130 well-preserved gills in 37 specimens of Vetulicola rectangulata (data source: Additional file 3) using PAST software [59], showing the correlation between gill slit diameter and gill pouch width (r: correlation coefficiency). [file 1741-7007-10-81-S2.TIFF]

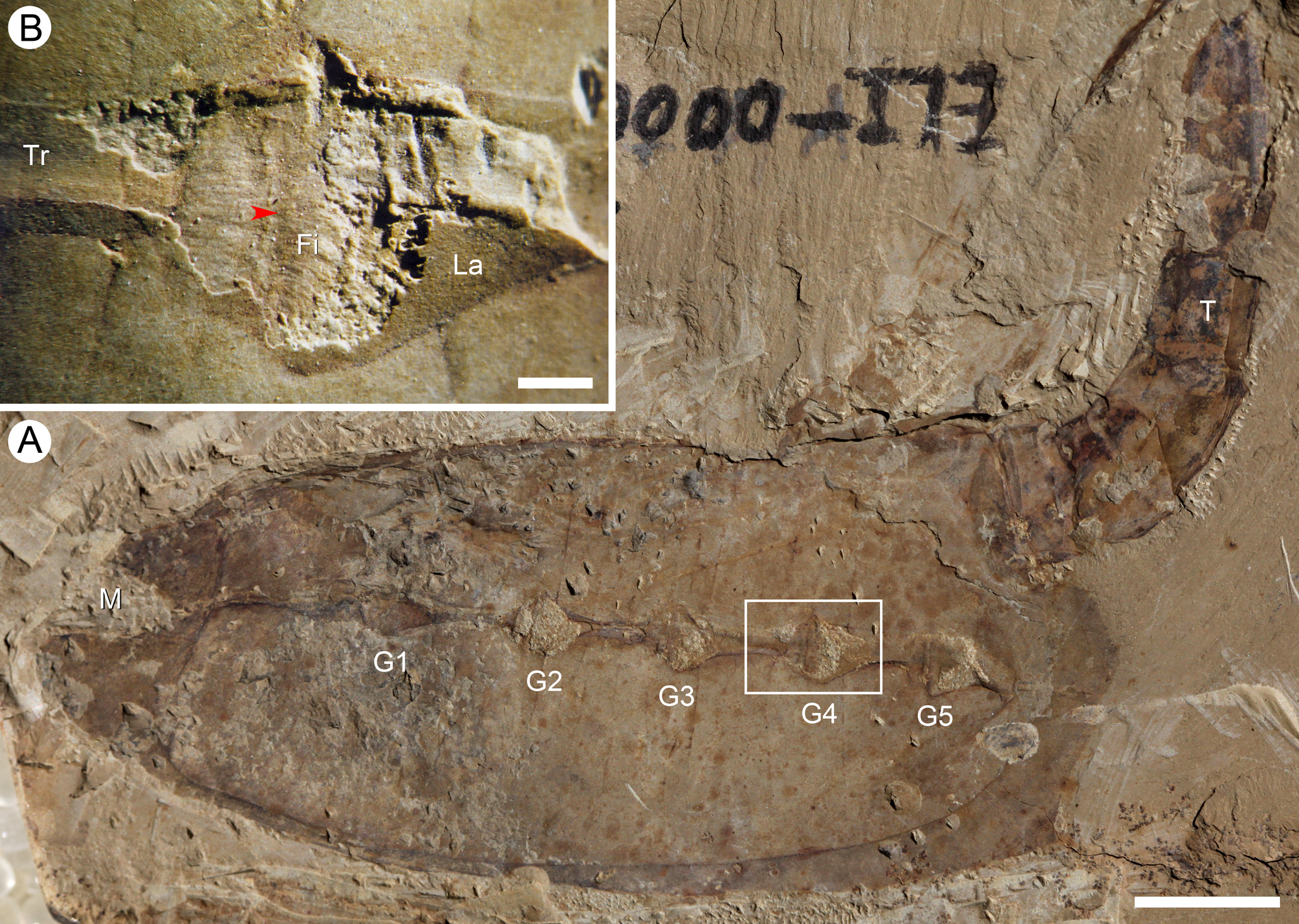

Supplement: Additional file 4 — Gill filaments in Vetulicola cuneata from Yunnan, China. (A) Laterally preserved specimen ELI-0000216. (B) Close-up image of the boxed area in (A), displaying tufts of filaments lining the surface of the gill pouch (viewed from the exterior). Transverse, slit-like gill opening denoted by arrowhead. Abbreviations: Fi, filaments; G1-5, gills 1 to 5; La, lappet; M, mouth; T, tail; Tr, trough. Scale bars: 1 cm in (A), (B); 5 mm in (C) to (F). [file 1741-7007-10-81-S4.JPEG]
