# Supplementary material for: Evidence for gill slits and a pharynx in Cambrian vetulicolians: implications for the early evolution of deuterostomes
Source: BMC Biol. 2012 Oct 2;10:81. doi: 10.1186/1741-7007-10-81 (PMC3517509; doi:10.1186/1741-7007-10-81)
Supplement: Additional file 3 — Morphometrics of vetulicolian gills. Morphometrics of vetulicolian gills given in mm. [file 1741-7007-10-81-S3.DOC]

**Table S1 Morphometrics of vetulicolian gills (mm).**

| **No.** | **Taxon** | **Specimen** | **Gill 1** | | | | **Gill 2** | | | | **Gill 3** | | | | **Gill 4** | | | | **Gill 5** | | | |
| --- | --- | --- | --- | --- | --- | --- | --- | --- | --- | --- | --- | --- | --- | --- | --- | --- | --- | --- | --- | --- | --- | --- |
|  |  |  | **L.** | **S.D.** | **P.W.** | **P.H.** | **L.** | **S.D.** | **P.W.** | **P.H.** | **L.** | **S.D.** | **P.W.** | **P.H.** | **L.** | **S.D.** | **P.W.** | **P.H.** | **L.** | **S.D.** | **P.W.** | **P.H.** |
| 1 | *Vetulicola rectangulata* | ELEL-EJ080255A | 7.96 | 2.28 |  | 2.82 | 8.47 | 2.39 |  | 2.97 | 7.51 | 2.04 |  | 2.50 | 6.21 |  |  | 2.23 |  |  |  |  |
| 2 | *V. rectangulata* | ELEL-EJ081561A | 7.55 |  |  | 3.07 | 7.73 | 1.94 |  | 3.41 | 7.55 | 1.68 |  | 3.23 | 6.74 | 1.56 |  | 2.89 | 10.60 |  |  | 1.95 |
| 3 | *V. rectangulata* | ELEL-EJ081952 | 8.33 |  |  | 3.82 | 7.16 |  |  | 3.86 | 8.01 |  |  | 3.52 | 6.49 |  |  | 2.57 |  |  |  |  |
| 4 | *V. rectangulata* | ELEL-EJ080158 | 8.12 | 5.15 | 6.65 | – | 9.46 | 5.72 | 6.76 | – | 13.13 | 5.10 | 6.67 | – | 7.28 | 3.92 | 5.18 | – |  |  |  | – |
| 5 | *V. rectangulata* | ELI-1027A | 6.71 | 3.25 | 4.21 | – | 8.16 | 4.00 | 5.58 | – | 7.96 | 2.89 | 4.89 | – | 7.16 | 2.19 | 5.24 | – | 12.15 |  | 4.03 | – |
| 6 | *V. rectangulata* | ELI-1033A | 8.99 | 2.48 | 3.95 | – | 10.21 | 3.44 | 5.08 | – | 8.70 | 3.01 | 5.07 | – | 7.50 | 2.58 | 4.50 | – | 12.42 | 2.26 | 4.63 | – |
| 7 | *V. rectangulata* | ELI-1036A | 9.52 | 3.52 | 4.72 | – | 10.67 | 4.13 | 6.30 | – | 9.56 | 3.73 | 5.83 | – | 8.09 | 3.76 | 4.94 | – |  |  |  | – |
| 8 | *V. rectangulata* | ELI-1037A | 10.21 | 3.47 | 5.20 | – | 10.95 | 3.66 | 5.68 | – | 9.45 | 3.88 | 5.90 | – | 8.34 | 3.36 | 5.28 | – | 11.98 | 3.18 | 5.21 | – |
| 9 | *V. rectangulata* | ELI-1121A | 9.89 | 1.53 | 4.09 | – | 10.63 | 3.30 | 4.88 | – | 9.19 | 3.34 | 4.97 | – | 8.32 | 2.96 | 4.67 | – | 12.11 | 2.64 | 4.18 | – |
| 10 | *V. rectangulata* | ELI-1070A | 6.27 | 2.20 | 3.34 | – | 6.89 | 1.96 | 3.57 | – | 5.61 | 1.71 | 3.53 | – | 4.41 | 1.60 | 3.16 | – | 8.34 | 1.36 | 3.29 | – |
| 11 | *V. rectangulata* | ELEL-SJ081080 | 6.98 |  | 3.32 | – | 7.05 | 2.79 | 3.78 | – | 6.28 | 2.50 | 3.40 | – | 6.00 | 2.84 | 3.82 | – | 10.28 |  | 3.02 | – |
| 12 | *V. rectangulata* | ELEL-EJ080339 | 8.60 |  | 4.27 | – | 8.77 |  | 4.56 | – | 8.59 | 3.21 | 5.19 | – | 6.26 | 2.62 | 4.48 | – | 10.9 | 2.17 | 4.41 | – |
| 13 | *V. rectangulata* | ELI-1015B | 8.03 | 4.27 | 5.51 | – | 8.57 | 4.58 | 6.82 | – | 7.88 | 4.56 | 6.98 | – | 7.43 | 3.96 | 5.68 | – | 8.81 | 2.98 | 4.60 | – |
| 14 | *V. rectangulata* | ELI-1088 | 6.45 | 2.48 |  | 3.48 | 7.65 | 2.88 |  | 3.86 | 8.13 |  |  | 3.71 |  |  |  |  |  |  |  |  |
| 15 | *V. rectangulata* | ELI-1081 | 5.94 | 1.40 | 3.38 | – | 6.24 | 2.13 | 3.87 | – | 5.45 | 1.67 | 3.97 | – | 4.35 | 2.04 | 3.57 | – | 4.50 |  | 3.45 | – |
| 16 | *V. rectangulata* | ELI-1054A | 8.69 | 3.34 | 6.26 | – | 10.24 | 3.54 | 7.54 | – | 8.96 | 3.13 | 6.26 | – |  |  |  | – |  |  |  | – |
| 17 | *V. rectangulata* | ELI-1004A | 8.84 | 2.33 | 4.33 | – | 9.26 | 4.07 | 5.22 | – | 8.80 | 4.07 | 5.37 | – | 7.05 | 4.12 | 5.48 | – | 7.07 | 3.36 | 4.82 | – |
| 18 | *V. rectangulata* | ELI-1001A | 9.03 |  | 5.26 | – | 8.64 |  | 5.19 | – | 7.50 |  | 6.07 | – | 6.17 | 3.40 | 5.59 | – | 9.35 | 2.79 | 5.63 | – |
| 19 | *V. rectangulata* | ELI-1013B | 6.02 | 1.41 | 4.16 | – | 8.74 | 1.34 |  | – | 7.82 | 1.96 | 4.27 | – | 6.79 | 2.01 | 3.54 | – |  |  | 2.50 | – |
| 20 | *V. rectangulata* | ELI-SS004A | 8.74 | 3.31 | 4.50 | – | 9.07 | 3.70 | 5.47 | – | 8.05 | 3.29 | 4.74 | – | 7.17 | 2.99 | 4.24 | – | 12.12 | 2.26 | 3.40 | – |
| 21 | *V. rectangulata* | ELI-SJ1159A | 9.03 | 3.52 | 5.04 | – | 8.70 | 3.71 | 5.44 | – | 7.38 | 3.83 | 5.89 | – | 7.70 | 2.45 | 5.25 | – | 11.38 | 2.30 | 4.48 | – |
| 22 | *V. rectangulata* | ELEL-SJ10UNK1 | 6.94 | 2.52 | 3.65 | – | 7.72 | 3.17 | 4.38 | – | 6.50 | 2.33 | 4.65 | – |  | 2.41 | 4.11 | – |  |  |  | – |
| 23 | *V. rectangulata* | ELEL-SJ10UNK2 | 7.72 | 2.70 | 4.32 | – | 8.27 | 3.32 | 5.27 | – | 7.36 | 3.08 | 5.13 | – | 6.02 | 2.55 | 4.66 | – | 9.14 | 1.37 | 3.60 | – |
| 24 | *V. rectangulata* | ELEL-SJ101975A | 7.01 | 1.96 | 4.58 | – | 7.83 | 2.26 | 5.30 | – | 6.81 | 2.40 | 4.84 | – | 5.87 | 1.84 | 4.15 | – | 7.76 | 1.38 | 3.59 | – |
| 25 | *V. rectangulata* | ELEL-SJ081642B | 7.33 | 2.00 | 3.43 | – | 7.56 | 2.02 | 3.91 | – | 7.2 | 2.27 | 3.79 | – | 6.70 | 2.11 | 4.28 | – | 12.63 | 1.69 | 3.42 | – |
| 26 | *V. rectangulata* | ELI-1118 | 8.12 | 2.50 | 3.87 | – | 8.55 | 2.62 | 4.02 | – | 7.25 | 2.80 | 4.34 | – | 6.80 | 2.91 | 4.76 | – | 10.63 | 2.41 | 4.29 | – |
| 27 | *V. rectangulata* | ELI-EJ05832 |  |  |  | – | 7.73 | 1.75 | 3.51 | – | 7.95 | 2.06 | 3.54 | – | 6.64 | 2.11 | 3.34 | – | 11.16 | 1.42 | 2.69 | – |
| 28 | *V. rectangulata* | ELI-SJ0605A | 10.51 | 2.25 | 3.45 | – | 8.06 | 2.39 | 3.28 | – | 8.02 | 2.33 | 3.21 | – | 6.46 | 2.00 | 3.19 | – | 20.21 | 1.49 | 2.47 | – |
| 29 | *V. rectangulata* | ELI-SJ1156A | 8.60 | 3.05 | 4.36 | – | 7.45 |  |  | – | 6.24 |  |  | – | 5.55 | 1.94 | 4.10 | – | 9.72 | 1.07 | 3.47 | – |
| 30 | *V. rectangulata* | ELI-1014A | 7.50 | 2.64 | 3.48 | – | 8.39 | 2.09 | 3.43 | – | 7.59 | 1.86 | 3.42 | – |  |  |  | – |  |  |  | – |
| 31 | *V. rectangulata* | ELI-1023A |  |  |  | – | 6.95 | 3.38 | 5.71 | – | 8.17 | 2.85 | 5.75 | – | 6.82 | 2.59 | 4.04 | – | 8.80 | 1.31 | 3.65 | – |
| 32 | *V. rectangulata* | ELEL-SJ101086B | 8.75 | 3.22 | 5.578 | – | 10.54 | 3.86 | 6.18 |  | 7.92 | 2.76 | 4.84 | – | 9.01 | 3.16 | 5.00 | – | 11.94 | 2.70 | 5.00 | – |
| 33 | *V. rectangulata* | ELI-1007A | 6.78 |  |  | 2.64 | 7.65 |  |  | 2.75 | 6.93 |  |  | 2.97 | 5.21 |  |  |  | 8.29 |  |  |  |
| 34 | *V. rectangulata* | ELI-SJ1158A | 7.21 | 1.96 | 3.52 | – | 8.74 | 2.24 | 3.87 | – | 7.39 | 2.31 | 4.20 | – | 6.90 | 2.10 | 4.34 | – | 12.01 | 2.21 | 3.70 | – |
| 35 | *V. rectangulata* | ELEL-EJ080244A | 6.30 |  |  | – | 8.13 | 2.51 | 4.38 | – | 10.20 | 2.33 | 4.25 | – |  | 2.68 |  | – |  |  |  | – |
| 36 | *V. rectangulata* | ELI-UNK3 | 6.84 | 1.52 | 3.43 | – | 7.09 | 1.89 | 3.70 | – | 6.61 | 2.12 | 3.79 | – | 3.80 | 1.68 | 3.39 | – | 9.36 | 1.67 | 3.02 | – |
| 37 | *V. rectangulata* | ELI-UNK4 | 3.29 |  | 2.25 | – | 4.90 |  | 2.16 | – | 3.57 |  | 2.54 | – | 3.19 |  | 2.36 | – | 5.88 |  | 1.96 | – |
| 38 | *V. monile* | ELI-1122B | 9.89 | 4.46 | 6.40 | – | 10.79 | 3.61 | 7.20 | – |  | 4.49 | 6.66 | – |  |  |  | – |  |  |  | – |
| 39 | *V. monile* | ELEL-SJ10UNK5 | 6.63 | 1.10 | 2.25 | – | 6.96 | 1.56 | 2.63 | – | 5.81 | 1.05 | 2.66 | – | 4.94 | 1.03 | 2.37 | – | 8.34 | 1.07 | 1.90 |  |
| 40 | *V. cuneatus* | ELI-0000214 | 10.97 | 3.60 | 5.99 | – | 9.84 | 4.04 | 6.30 | – | 8.57 | 4.67 | 6.83 | – | 7.13 | 3.11 | 5.35 | – | 12.03 | 2.21 | 4.52 |  |
| 41 | *V. cuneatus* | ELI-0000215 | 4.99 | 2.30 | 3.35 | – | 5.35 | 2.25 | 3.58 | – | 4.52 | 1.58 |  | – | 4.13 | 1.67 |  | – | 6.22 |  |  | – |
| 42 | *V. cuneatus* | ELI-0000218 | 6.99 |  | 3.57 | – | 7.44 |  | 4.19 | – | 6.24 |  | 3.76 | – | 5.12 |  | 4.18 | – | 9.00 |  | 3.53 | – |
| 43 | *V. cuneatus* | ELI-0000207-2 | 7.86 | 2.96 | 4.20 | – | 7.49 | 3.04 | 4.33 | – | 7.09 |  | 3.66 | – | 6.72 |  | 2.91 | – | 8.83 |  |  | – |
| 44 | *V. cuneatus* | ELI-0000207-1 | 7.33 |  | 4.38 | – | 8.49 |  | 4.49 | – | 7.07 |  | 4.68 | – | 5.78 |  | 4.33 | – |  |  | 3.62 | – |
| 45 | *V. cuneatus* | ELI-0000320 | 6.39 | 2.02 | 3.49 | – | 6.46 | 2.02 | 3.55 | – | 6.56 | 2.11 | 3. 83 | – | 4.93 |  | 3.29 | – | 8.56 |  |  | – |
| 46 | *Didazoon haoae* | ELEL-SJ081389 | 7.18 | 4.30 | 6.59 | – | 9.01 |  | 6.02 | – | 9.61 |  | 8.56 | – | 8.34 |  | 9.96 | – | 8.27 |  | 7.14 | – |
| 47 | *D. haoae* | ELI-JS1001A | 4.78 |  | 5.01 | – | 7.68 |  | 6.10 | – | 7.21 |  | 6.59 | – | 6.42 |  | 6.13 | – |  |  |  | – |
| 48 | *D. haoae* | ELI-2001A | 6.95 |  | 6.53 | – | 9.57 |  | 9.29 | – | 9.75 |  | 9.80 | – | 7.72 |  | 8.56 | – | 5.66 |  | 6.35 | – |
| 49 | *D. haoae* | ELI-1006A |  |  |  | – | 9.54 |  | 8.02 | – | 7.15 |  | 7.62 | – | 7.55 |  | 7.30 | – | 6.62 |  | 5.47 | – |
| 50 | *D. haoae* | ELI-2010A | 7.02 | 2.66 | 4.34 | – | 8.83 | 4.06 | 7.50 | – | 9.81 | 5.37 | 7.77 | – | 8.46 | 3.09 | 5.53 | – | 11.12 | 2.16 | 3.43 | – |
| 51 | *D. haoae* | ELI-SJ2106A | 7.83 |  | 6.04 | – | 7.26 |  | 8.30 | – | 6.70 |  | 8.10 | – | 8.59 |  | 7.95 |  | 15.38 |  | 6.59 | – |
| 52 | *D. haoae* | ELI-SJ2107A | 7.08 |  | 4.40 | – | 9.87 |  | 7.39 | – | 10.67 |  | 7.65 | – |  |  |  | – |  |  |  | – |
| 53 | *D. haoae* | ELEL-SJ101067A | 5.21 |  | 3.26 | – | 6.85 |  | 6.11 | – | 8.14 |  | 5.52 | – | 7.36 |  | 4.84 | – | 12.54 |  | 5.08 | – |
| 54 | *D. haoae* | ELEL-SJ101095A | 9.95 |  | 5.61 | – | 10.89 |  | 9.70 | – | 9.98 |  | 9.70 | – | 9.34 |  |  | – | 16.98 |  | 8.73 | – |
| 55 | *D. haoae* | ELI-0000198 | 4.23 |  | 5.03 | – | 6.46 |  | 7.18 | – | 6.60 |  | 7.07 | – | 6.43 |  | 6.07 | – | 11.19 |  | 4.60 | – |
| 56 | *D. haoae* | ELEL-SJ081138A | 7.71 |  | 4.11 | – | 11.06 |  | 6.25 | – | 10.55 |  | 7.34 | – | 6.90 |  | 6.81 | – | 15.66 |  | 6.57 | – |
| 57 | *Pomatrum ventralis* | YKLP 10914a | 5.44 |  | 3.65 | – | 7.98 |  | 7.23 | – | 9.75 |  | 8.96 | – | 7.90 |  | 7.65 | – | 6.33 |  | 6.31 | – |
| 58 | *Yuyuanozoon magnificissimi* | CFM00059 | 14.80 |  |  | – | 21.82 | 11.05 | 18.17 | – | 19.47 |  | 15.92 | – | 20.52 |  | 12.35 | – | 19.72 |  | 10.51 | – |

Inapplicable measurements, each represented by a hyphen (–), are precluded by the orientation or taphonomy of individual specimens.

Abbreviations: L., length; P.H., height of gill pouch; P.W., width of gill pouch; S.D., diameter of gill slit.
